# Supplementary material for: Morphological variations of the femoral head-neck junction in historical skeletal material
Source: PeerJ. 2025 Oct 16;13:e20236. doi: 10.7717/peerj.20236 (PMC12535743; doi:10.7717/peerj.20236)
Supplement: Supplemental Information 2 [file peerj-13-20236-s002.docx]

**Supplementary material**

**Title:** Femoral head-neck junction changes in skeletal material

**Authors:** Anna Myszka; Anna Maria Kubicka*;

^1^Institute of Biological Science, Cardinal Stefan Wyszynski University in Warsaw, Poland

^2^Department of Zoology, Poznan University of Life Sciences, Poland

^3^PaleoFED Team, UMR 7194 Histoire Naturelle de l'Homme Préhistorique, CNRS, Département Homme et Environnement, Muséum national d'Histoire naturelle, Paris, France

*Corresponding author

Figures: 1

| 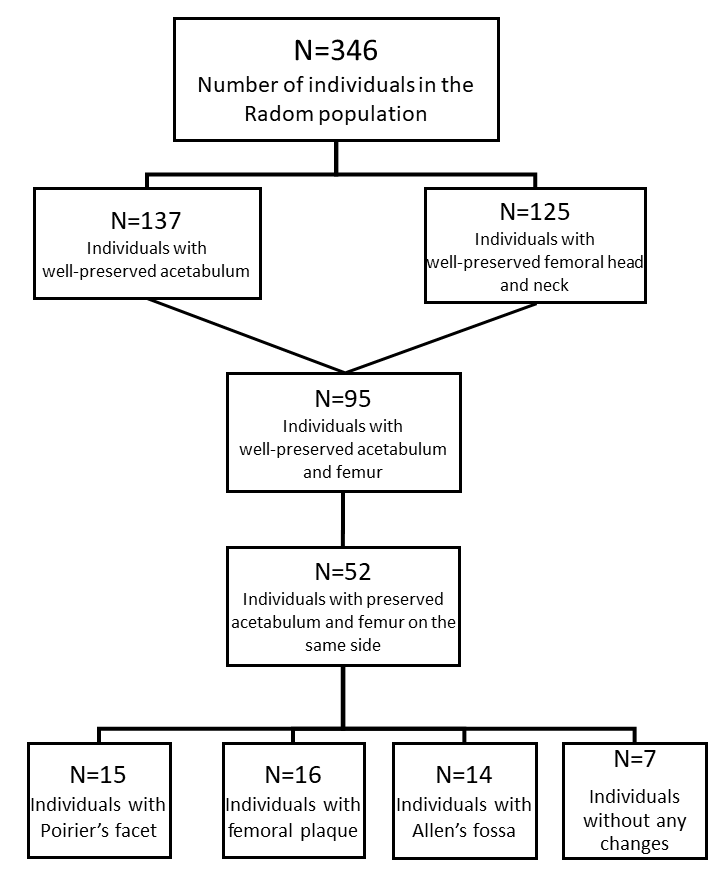 |
| --- |
| Figure S1. Flowchart showing the selection of bones for analysis |
